# An IS element-driven antisense RNA attenuates the expression of serotype 2 fimbriae and the cytotoxicity of *Bordetella pertussis*

**DOI:** 10.1080/22221751.2025.2451718

**Published:** 2025-01-09

**Authors:** Alexandre D’Halluin, Denisa Petráčková, Ivana Čurnová, Jakub Držmíšek, Jan Čapek, Peggy Bouquet, Loïc Henin, Rudy Antoine, Loïc Coutte, Camille Locht, Branislav Večerek, David Hot

**Affiliations:** a U1019 – UMR8204 – CIIL - Center for Infection and Immunity of Lille, Univ. Lille, CNRS, Inserm, CHU Lille, Institute Pasteur de Lille, Lille, France; b Laboratory of Post-Transcriptional Control of Gene Expression, Institute of Microbiology of the Czech Academy of Sciences, Prague, Czech Republic; c Univ. Lille, CNRS, Inserm, CHU Lille, Institut Pasteur de Lille, US 41 - UAR 2014 - PLBS, F-59000, Lille, France

**Keywords:** *Bordetella pertussis*, antisense RNA, insertion sequence, fimbriae serotype 2, modulation of virulence, cytotoxicity towards macrophages

## Abstract

Insertion sequences (IS) represent mobile genetic elements that have been shown to be associated with bacterial evolution and adaptation due to their effects on genome plasticity. In *Bordetella pertussis*, the causative agent of whooping cough, the numerous IS elements induce genomic rearrangements and contribute to the diversity of the global *B. pertussis* population. Previously, we have shown that the majority of IS-specific endogenous promoters induce the synthesis of alternative transcripts and thereby affect the transcriptional landscape of *B. pertussis*. Here, we describe the regulatory RNA Rfi2, which is transcribed from the P_out_ promoter of the IS*481* gene *BP1118* antisense to the adjacent *fim2* gene encoding the major serotype 2 fimbrial subunit of *B. pertussis*. Among the classical bordetellae, Rfi2 is unique to *B. pertussis*, suggesting its specific role in virulence. We show that Rfi2 RNA attenuates *fim2* transcription and, consequently, the production of the Fim2 protein. Interestingly, the mutant that does not produce Rfi2 displayed significantly increased cytotoxicity towards human macrophages compared to the parental strain. This observation suggests that the Rfi2-mediated reduction in cytotoxicity represents an evolutionary adaptation of *B. pertussis* that fine-tunes its interaction with the human host. Given the immunogenicity of Fim2, we further hypothesize that Rfi2-mediated modulation of Fim2 production contributes to immune evasion. To our knowledge, Rfi2 represents the first functionally characterized IS element-driven antisense RNA that modulates the expression of a virulence gene.

## Introduction

Genomic rearrangements are important drivers of pathogen adaptation to the host environment, antibiotic pressure, and vaccine-induced immunity [1,2]. In bacteria, genome rearrangements are predominantly caused by homologous recombination between two regions displaying high sequence similarity, such as insertion sequences (IS). These mobile genetic elements, encoding a transposase, are flanked by terminal inverted repeats that allow their excision and site-specific insertion into the host genome [2]. While insertion of an IS into a gene usually results in its inactivation, recombination between IS elements leads to genome decay by deletion or reshuffling of parts of the genome. Consequently, changes in gene order and genomic rearrangements can alter the global expression profiles [2,3]. IS elements can also directly affect the expression profile of their adjacent genes through the activity of IS-specific internal promoters (P_in_ or P_out_), or by generating strong hybrid promoters (P_hyb_) [4–6]. Therefore, IS-specific genome rearrangements and the resulting IS-driven transcription can affect the global transcriptomic profiles and thereby contribute to strain diversity [7–9].

*Bordetella pertussis* is a Gram-negative strictly human pathogen of the respiratory tract and the etiological agent of whooping cough (pertussis). After infection, *B. pertussis* colonizes the ciliated epithelium of the human upper respiratory tract and causes inflammation, activation of immune responses and damage to host tissues [10]. To efficiently colonize the respiratory tract and evade the immune response, *B. pertussis* produces a variety of virulence factors, including toxins, such as adenylate cyclase and pertussis toxins, and adhesins such as filamentous haemagglutinin and fimbriae [11]. *B. pertussis* produces two serologically distinct types of fimbrial subunits Fim2 and Fim3, which are encoded by the *fim2* and *fim3* genes, respectively. Their expression is controlled by the length of a stretch of cytosine residues located between −10 and −35 promoter regions [12], so that *B. pertussis* can produce either Fim2, Fim3 or both fimbriae [13]. Furthermore, the expression of virulence factors, including fimbriae, is positively regulated by the two-component system BvgAS, which consists of the sensor kinase BvgS and the response regulator BvgA [14]. The BvgAS phosphorelay controls a spectrum of gene expression states that allow cells to switch between the virulent Bvg^+^ phase, the intermediate Bvg^i^ phase and the avirulent Bvg^-^ phase. The signals sensed by BvgS in the host airways remain unknown, but the levels of phosphorylated BvgA are strongly reduced in the presence of millimolar concentrations of nicotinic acid (20 mM) or sulphate ions (50 mM) [15,16], while lower temperature (24°C) results in only a partial loss of BvgA phosphorylation [17].

In recent decades, despite high vaccination coverage, a resurgence of the whooping cough has been observed, making it one of the most prevalent vaccine-preventable diseases [18,19]. The growing number of pertussis cases has been associated with incomplete and short-lived immunity induced by currently used acellular vaccines, asymptomatic transmission, and adaptation of the pathogen to vaccine pressure [2,20–23]. The genome of *Bordetella pertussis*, the causative agent of whooping cough, contains more than 240 IS elements, mostly IS*481*, and their rearrangements have been proposed as one of the evolutionary forces enabling adaptation to vaccine pressure [24,25]. Indeed, global use of vaccines has led to the emergence of strains lacking antigens included in the current acellular vaccine, such as pertactin, filamentous haemagglutinin and pertussis toxin [26–29]. In most of these isolates, IS*481*-specific recombination events have led to deletion of the corresponding genomic region or the genes were disrupted by direct insertion of the IS*481* element. In our previous study, we have shown that almost all IS elements in *B. pertussis*, and IS*481* in particular, initiate the synthesis of transcripts oriented in the sense or antisense direction of their flanking genes [7]. The polar effect of an IS-specific transcript on the expression of a downstream gene, transcribed in the same direction has already been documented in *B. pertussis* for the *katA* and *bteA* genes [30,31]. However, to our knowledge, the regulatory effect of an IS-driven antisense transcript on the expression of a neighbouring gene has not yet been described.

In our previous work, we identified several regulatory RNAs that are transcribed antisense to genes encoding virulence factors (asRNAs) [7]. Interestingly, one of these seemed to be expressed from the P_out_ promoter of an IS*481* element. In this work, we investigated this asRNA called Rfi2 (repressor of *fim2*), which is transcribed from the P_out_ promoter of the IS*481* transposase gene *BP1118* in the antisense direction to the *B. pertussis fim2* gene. Due to its high immunogenicity, Fim2 is a component of some acellular vaccines [32]. We show here that IS*481*-driven Rfi2 expression impacts on expression of *fim2* and reduces the cytotoxicity of the pathogen towards human phagocytic cells. This may consequently affect the virulence of *B. pertussis* and its interaction with the host during infection.

## Materials and methods

### Bacterial strains and culture conditions

All strains and plasmids used in this study are listed in Table S1. *Bordetella* strains were grown on Bordet-Gengou Agar plates supplemented with 10% defibrinated sheep blood and 100 µg/ml streptomycin at 37°C for 48 h. For liquid cultures, bacteria were cultivated in modified Stainer Scholte (SS) medium [33] supplemented with 0.1% cyclodextrin and 1% casamino acids (Difco) at 37°C. To harvest samples for RNA and protein isolation, *Bordetella* cells were grown overnight in modified SS medium to late exponential phase (OD_600_ ≈ 2.0). For phenotypic modulation, strains were grown in modified SS medium supplemented with 50 mM MgSO_4_ (hereafter referred to as sulphate).

### Construction of the mutants

*B. pertussis* mutants were generated by homologous recombination in the BPSM strain [34], a derivative of the Tohama I strain, which is referred to as the wild-type (wt) in this study. To construct the Δ*rfi2* strain, two DNA fragments of 300pb corresponding to the upstream and downstream regions flanking the deletion site, were produced by custom DNA synthesis at Integrated DNA Technologies (https://eu.idtdna.com) and inserted into the *Bam*HI and *Hin*dIII sites of pJQ200-mp18-*rplS* . The resulting plasmid was used to transform *Escherichia coli* SM10 and then transferred to *B. pertussis* strains by conjugation. After two recombination events, the strain carrying the desired mutation was selected. The absence of secondary mutations was verified by complete genome sequencing of the strains on Illumina Nextseq, 500 using the Nextera XT DNA Library Preparation Kit (Illumina). The Δ*rfi2* strain contains a 1053-pb deletion of *BP1118*. The terminator 2039/2040 of the *greB* gene from *B. bronchiseptica* RB50 [35], predicted by the TransTermHP software, was introduced downstream of the *greB* gene to prevent read-through transcription from *greB* into *fim2*.

The strain that overexpresses Prfi2 was generated as follows. An 800-bp DNA fragment containing the 5’-UTR of the *BP1118* transposase gene including promoters P_out_ and P_in_ and the *fim2* gene lacking its promoter region was synthesized at Integrated DNA Technology. The *fim2* promoter was deleted as to avoid overlapping *fim2* transcription and possible attenuation of Rfi2 expression. The DNA fragment was inserted between the *Hin*dIII and *Bam*HI sites of pBBR1MCS [36]. This multicopy and non-integrative plasmid was introduced into *Escherichia coli* SM10 and then transferred into *B. pertussis* Δ*rfi2* by conjugation, allowing ectopic expression of Rfi2 from the plasmid and endogenous expression of *fim2*.

### RNA extraction and Northern blot analysis

*Bordetella* cells grown to exponential phase in modified SS medium were pelleted by centrifugation for 8 min at 2,800 x *g* and stored at −80°C. Cell pellets were suspended in TE buffer (10 mM Tris, 1 mM EDTA; pH 8.0) containing 1 mg/ml lysozyme (Sigma-Aldrich), and total RNA was isolated from the lysed cells by using TRI Reagent (Sigma) according to the manufacturer’s protocol. DNA was removed from RNA samples by treatment with DNase I Kit (Sigma-Aldrich). The quantity and quality of RNA were checked using Nanodrop 2000 (Thermo Fisher) and Bioanalyzer 2100 (Agilent Technology). Total RNA (5 µg per lane) was mixed with 2X RNA loading dye and denatured for 4 min at 70°C before electrophoretic separation on a 10% acrylamide:bis-acrylamide (37.5:1) gel prepared in 0.5X Tris-Borate-EDTA buffer (TBE) containing 8M urea. The RNA was then electro-transferred onto BrightStar Plus (Thermo Fischer) nylon membranes and crosslinked by UV light. The membrane was hybridized overnight at 55°C with biotinylated probes specific for Rfi2, *fim2* and 5S rRNA transcripts. Biotinylated RNAs transcribed *in vitro* using RNA Century™ Marker Template (Invitrogen) served as size markers. Blots were developed using chemiluminescent detection with the BrightStar® BioDetect™ kit (Ambion).

### Identification of transcription start sites by 5’ RACE

Transcription start sites (TSSs) were determined by using Deep 5’ RACE as described [37]. Briefly, 30 µg of total RNA was treated with 1 U of 5’ terminator exonuclease (Epicentre) for 1 h at 37°C, purified by organic extraction and treated with 1 U of 5’ RNA polyphosphatase (Epicentre) for 1 h at 37°C. A synthetic RNA adapter was ligated by using 40 U of RNA T4 ligase (New England Biolabs) for 1 h at 37°C, followed by organic extraction. RNA was reverse transcribed using the SuperScript III Kit (Thermo Fisher) and random oligonucleotides (Thermo Fisher) before PCR amplification using the Herculase Fusion II Kit (Agilent). The primers are listed in Supplementary Table S2. Oligonucleotides A and P1 were ligated to the amplified cDNA by using the Ion Plus Fragment Library Kit (Thermo Fisher). The cDNA was sequenced on the PGM sequencing machine (Life Technology). Reads were trimmed with Cutadapt software and aligned on the Tohama I genome NC_002929.2 with CLC Genomics Workbench software.

### Determination of RNA stability

The *B. pertussis* strains were cultured in modified SS medium in the absence or presence of 50 mM sulphate. At an OD_600_ ≈ 2, rifampicin (100 µg/ml) was added to the cultures and cells were harvested at different time points after the addition of rifampicin and pelleted by centrifugation. Total RNA was extracted by using TRI Reagent (Sigma) and subjected to Northern blot analysis as described above. The levels of Rfi2, *fim2* and 5S rRNA transcripts were calculated by using the Scion Image v4.0 software (https://scion-image.software.informer.com/4.0/). All experiments were carried out in duplicate.

### Analysis of RNA interactions by electrophoretic mobility shift assay (EMSA)

Rfi2 (280 nt), the 3’end of *fim2* (218 nt) and the 5’ end of *BP1118* (108 nt) were transcribed *in vitro* by using the Herculase Fusion II Kit (Agilent) with 5’ primers carrying the T7 promoter sequence (TAATACGACTCACTATAGGG). The primers are listed in Supplementary Table S2. The PCR fragments were inserted into pCR2.1-TOPO using the TOPO-TA cloning Kit (Thermo Fisher). The plasmids were introduced into *E. coli* DH5α and used as a template for an additional amplification and purification. RNA was transcribed for 2 h at 37°C by using Ampliscribe T7-Flash Biotin-RNA (Epicentre) following the manufacturer’s recommendations. Rfi2 was biotinylated with 4.5 mM unlabelled and 3 mM labelled UTP. The PCR fragments were digested with 1U DNase for 15 min at 37°C. RNA was subjected to electrophoresis using a 6% acrylamide:bis-acrylamide gel (37.5:1; vol:vol) containing 8 M urea, and the gel slices containing the RNA were excised. The gel slices were incubated in 0.5 mM ammonium acetate (pH 5.2), 0.1% SDS, 1 mM EDTA, 0.1% phenol overnight at 37°C, and the RNA was purified by organic extraction. Hybridization reactions were carried out with 5 nM biotinylated Rfi2 and 5 nM of each target and 5 nM non-biotinylated Rfi2 as competitors, where indicated. Transcripts were denatured for 2 min at 70°C in 10 mM Tris-HCl (pH 8.0), 50 mM NaCl, 50 mM KCl and 10 mM MgCl_2_ and then incubated at 37°C for 30 min. Samples were subjected to electrophoresis on a non-denaturing 5% acrylamide:bis-acrylamide gel (37.5:1; vol:vol) for 80 min at 4°C in TBE. The EMSA was then developed as described above.

### Western blot analysis

*B. pertussis* cells were grown in modified SS medium at 37°C and cell pellets from 3-ml aliquots were suspended in sample buffer composed of 50 mM Tris-HCl, pH 8.0, protease inhibitor cocktail (Sigma Aldrich) and 10 µg/ml DNase I. Cells were lysed using a French Press cell (Thermo Fisher) with three cycles at 1000 Pa and then centrifuged at 2800 × *g* for 15 min at 4°C. Proteins in the supernatant were mixed with Laemmli buffer (Sigma Aldrich) and incubated at 95°C for 10 min. Samples equivalent to 0.1 OD_600_ were separated on 12.5% SDS-polyacrylamide gels and electro-transferred onto nitrocellulose membrane Hybond-C extra (GE Healthcare). Membranes were blocked with 10% skim milk and probed with a rabbit anti-Fim2 antibody (Alpha Diagnostic International, #FIM-25S) at a 1:5,000 dilution, followed by incubation with an anti-rabbit IgG secondary antibody conjugated to horseradish peroxidase (Thermo Fisher) at a dilution of 1:20,000. The blots were developed by using the Amersham ECL-Prime Kit and the Amersham Imager 680 (GE Healthcare). The quantity of Fim2 protein was quantified by using Quantity One software version 4.6.9 (BioRad).

### Amplification and sequencing of the *fim2* promoter region

*B. pertussis* cells were denatured at 90°C for 5 min and centrifuged at 13,000 rpm for 5 min. The cleared lysate (1 µl) was directly used as a template for a PCR reaction with *fim2*-specific primers and Herculase II Fusion polymerase (Agilent). First, the *fim2* promoter region was amplified with the primers fim2fw1 and fim2rev1, yielding a 349-bp DNA fragment. The fragment was gel-purified using the QIAquick Gel Purification Kit (Qiagen) and sequenced using the primers fim2fw2 and fim2rev2 in the Eurofins Genomics sequencing facility (https://eurofinsgenomics.eu/). Obtained electropherograms were visualized using SnapGene software (https://www.snapgene.com/).

### Conservation of the IS*481*/*fim2* locus in *B. pertussis*

The BLAST tool of the BIGSdb website of the Institute Pasteur (https://bigsdb.pasteur.fr/) was used to analyse recent isolates to determine whether IS*481* is located adjacent to the *fim2* gene in these strains. The analysis was restricted to strains from the *B. pertussis* phylogeny project in the Bordetella cgMLST database (https://bigsdb.pasteur.fr/cgi-bin/bigsdb/bigsdb.pl?db = pubmlst_bordetella_isolates). The query sequence was extracted from the Tohama I reference genome from coordinates 1,175,755 to 1,176,054, including the 5'UTR of *BP1118* and the 3’ end of *fim2* (*BP1119*). The BLASTN word size was 11 nucleotides and the BLASTN scoring was reward:2; penalty:−3; gap_open:5; gap_extend:2.

### Differentiation and infection of macrophages

Differentiation of THP-1 monocytes into THP-1 macrophages and their infection with *B. pertussis* cells were performed as described [38]. Briefly, THP-1 cells (ATCC; TIB-202) were grown in 48-well plates in Roswell Park Memorial Institute (RPMI) 1640 medium (Sigma, R8758) supplemented with 10% heat-inactivated fetal bovine serum (Sigma) at 37°C in a humidified incubator (5% CO_2_). For differentiation into macrophages, THP-1 monocytes were stimulated by addition of 100 nM phorbol 12-myristate 13-acetate (Sigma) for 72 h, followed by a 24-hour resting period in plain RPMI medium.

Differentiated THP-1 macrophages were infected with *B. pertussis* in RPMI medium and the multi-well plates were then centrifuged at 600 × *g* for 3 min to facilitate the interaction of the bacteria with the macrophages. After 1 h of incubation (37°C; 5% CO_2_), extracellular bacteria were removed by washing with RPMI medium, and the remaining bacteria were killed by incubation in RPMI medium containing 100 μg/ml of polymyxin B sulphate (Sigma) for 1 h (37°C; 5% CO_2_).

### Cell viability and cytotoxicity assays

The viability of infected THP-1 macrophages was determined spectrophotometrically as the capacity of mitochondrial dehydrogenase to induce cleavage of tetrazolium salt to formazan by using the WST-1 assay kit (Roche) according to the manufacturer’s protocol. Briefly, THP-1 monocytes were differentiated into macrophages in 48-well plates (4.6 × 10^5^ cells per well) and infected with *B. pertussis* at MOI 30 and 50. 2 h post-infection (1 h of incubation with the bacteria, followed by 1 h of polymyxin B treatment), infected cells were washed intensely with prewarmed RPMI medium and then incubated in 200 µl of RPMI medium and 20 µl of WST-1 substrate per well for 40 min at 37°C. In parallel, uninfected cells were treated in the same manner and served as controls (100% viability). The absorbance of the formazan dye measured at 450 nm correlates directly with the number of viable cells and was measured with a scanning multi-well spectrophotometer Epoch (BioTek).

Cytotoxicity of *B. pertussis* towards THP-1 cells was determined using the CellTox™ Green Cytotoxicity Assay (Promega) as previously described [38]. Briefly, this assay measures changes in membrane integrity that occur as a result of cell death. The fluorescent signal resulting from the binding of the dye to the dead-cell DNA is proportional to cytotoxicity. THP-1 monocytes seeded in 48-well plates (4.6 × 10^5^ cells per well) were differentiated in colourless RPMI medium (R7509, Sigma) supplemented with L-glutamine (0.03%) and infected by adding 100 µl of a *B. pertussis* cell suspension (containing 2.3 × 10^7^ cells; MOI 50). After infection, the cells were washed intensively with prewarmed RPMI medium, and the assay was started by adding the CellTox^TM^ reagent (0.1 µl) in 100 µl/well colourless RPMI according to the manufacturer’s protocol. Uninfected cells were treated in the same manner and served as controls. The macrophages were then incubated in a Tecan Spark multimode microplate reader (37°C, 5% CO_2_), and the fluorescence was measured at Ex/Em = 490 ± 10/520 ± 10 nm for 18 h after the addition of the reagent.

### Fluorescence microscopy

THP-1 monocytes were seeded in 48-well plates (4.6 × 10^5^ cells per well) and differentiated into macrophages in colourless RPMI medium. In parallel to cytotoxicity assays, macrophages were infected with *B. pertussis* cells at MOI 30 and washed twice with RPMI 4 h after infection. Uninfected macrophages served as controls. Cells were stained with the red fluorochrome propidium iodide (PI) (30 µM) and the green fluorochrome SYTO-9 (5 µM) for 15 min at room temperature in the dark. PI stains necrotic cells and its excess over the SYTO-9 fluorochrome ensures that necrotic, nonviable cells appear red and viable cells appear green. The macrophage cells were imaged at 10x magnification using Olympus motorized fluorescence microscope IX83 with cellSens software (Olympus LS). Pictures were processed with ImageJ 1.54d software (https://imagej.net/ij/index.html) and fluorescent cells were counted manually.

## Results

### Identification of an asRNA transcribed from the IS*481* transposase P_out_ promoter antisense to *fim2*

**Figure 1.**
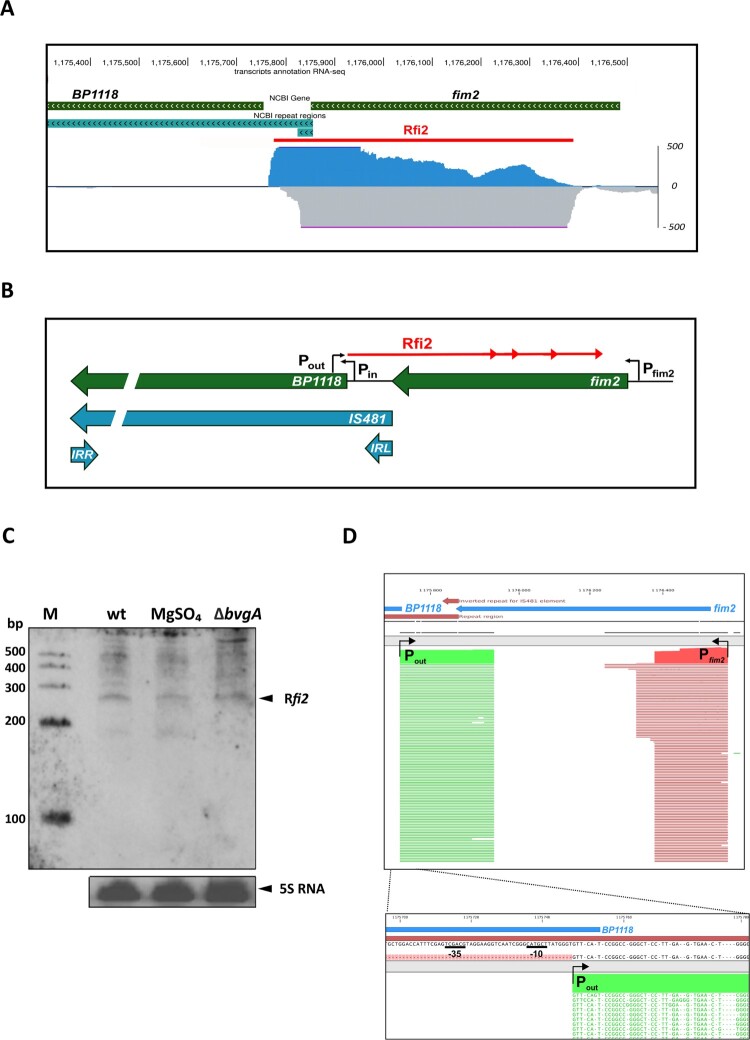
Characterization of Rfi2. (A) Visualization of the deep RNA-seq dataset showing the region between *BP1118* and *fim2* genes. The graphs show the sequencing depth of the positive (blue) and negative (grey) strands. The gene annotations are depicted as green arrows. The red bar denotes the genomic position of Rfi2 RNA. Data and hub screenshot were obtained from [7]. (B) Scheme of the transcriptional organization of the *BP1118-fim2* region as inferred from RNA-seq data. Black bent arrows indicate promoter regions. Green and blue bars indicate the annotated genes and the IS*481* element including its left (IRL) and right (IRR) inverted repeat regions, respectively. Red arrow indicates the Rfi2 transcript driven by P_out_ promoter, arrowheads illustrate the abortive termination of the Rfi2 transcription yielding several Rfi2-specific transcripts. (C) Northern blot analysis of Rfi2 expression. Rfi2 transcript was detected using a biotinylated probe hybridized with 5 µg of total RNA isolated from *B. pertussis* grown in the absence (wt) and presence of sulphate (MgSO_4_). In parallel, Rfi2 was detected also in total RNA isolated from the Δ*bvgA* strain. The signals corresponding to the Rfi2 transcript (upper panel) and the 5S rRNA (lower panel) are indicated by the black arrowheads. The biotinylated Century RNA ladder was loaded as a molecular size marker (M). (D) Determination of the transcription start site of Rfi2 using the 5’RACE method. Upper panel: alignment of Rfi2-specific and *fim2*-specific reads is shown in green and red, respectively*.* Black bent arrows indicate transcription start site of Rfi2 and *fim2* RNAs driven by corresponding promoter. Lower panel: detail of the Rfi2 promoter region. Black bent arrow indicates transcription start site of Rfi2. Plausible −10 and −35 boxes of the P_out_ promoter are underlined. Reads were aligned using CLC Genomics Workbench v10.

**Figure 2.**
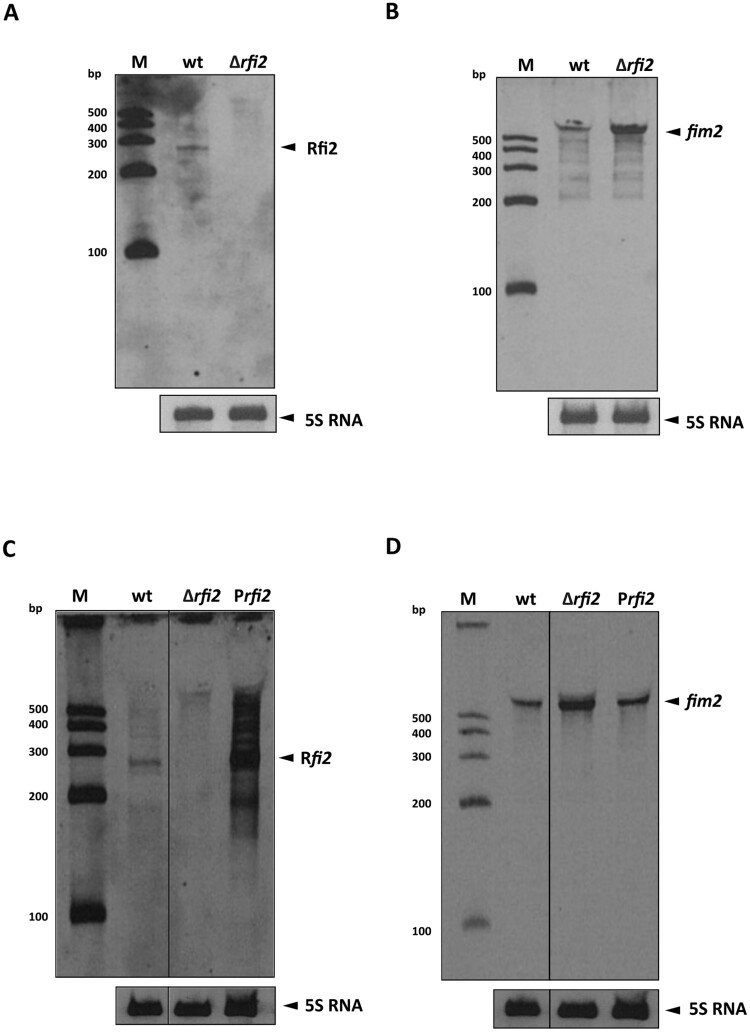
The effect of Rfi2 on *fim2* expression. Northern blot was performed using total RNA isolated from wt, Δ*rfi2,* and P*rfi2* strains hybridized with biotinylated probes specific for Rfi2 (A, C) or *fim2* (B, D) transcripts. The signals corresponding to detected Rfi2 and *fim2* transcripts (upper blots) and to 5S RNA (lower blots, loading controls) are indicated by black arrowheads. The biotinylated Century RNA ladder (M) was loaded as a molecular size marker.

In our previous study, we characterized the primary transcriptome of *B. pertussis* Tohama I and unveiled a long list of candidate regulatory non-coding RNAs [7]. Expression of some candidate transcripts resulted from transcription driven by the internal promoter P_out_ of IS*481*, located in the transposase gene. One of them, named Rfi2, is transcribed in the antisense orientation of *fim2*, as confirmed by dRNA-seq data [7], which showed that Rfi2 synthesis is initiated from P_out_ located within the IS*481 BP1118* open reading frame (Figure 1(A,B)). Bioinformatic analyses identified no terminator structure downstream of P_out_ (data not shown), suggesting that transcription could proceed in the antisense orientation of the neighbouring *fim2* gene. Using a probe derived from the *fim2* sequence, we determined the Rfi2-specific signals by Northern blot analysis. We detected several Rfi2-specific transcripts, including a prominent transcript of ≈ 280 nt (Figures 1(C) and 2(A,C)), suggesting that P_out_-driven transcription terminates abortively at several sites, as previously shown for IS*10* [39] and that Rfi2 represents several P_out_-driven transcripts, all of which are complementary to *fim2* mRNA. Since *fim2* belongs to virulence genes controlled by the BvgAS system, we wondered whether the expression of Rfi2 is also BvgAS-dependent. Therefore, we examined Rfi2 levels in the wt strain grown in the absence or presence of 50 mM MgSO_4_ and also in the Δ*bvgA* strain. Northern blot analysis revealed that the Rfi2 transcript is present at similar levels in all samples (Figure 1(C)), indicating that the expression of Rfi2 is not dependent on BvgAS.

The TSS of IS*481 BP1118*, *fim2* and Rfi2 were determined by 5’RACE. The TSS of Rfi2 is located 19 bp from the 5´-end of the *BP1118* transcript, creating an overlap, and is preceded by plausible −35 (TCGAGT) and −10 (CATGCT) sequences at an appropriate distance (Figure 1(D), Figure S1A). The TSS of *BP1118* transposase gene is preceded by −35 (TGGAAA) and −10 (TTCACT) regions (Figure S1A, B). The complementarity of 19 nt between P_in_- and P_out_-driven transcripts of IS*481* is similar to the overlap of 35 nt between the RNA-IN and RNA-OUT of *E. coli* IS*10* element [40] (Figure S1A). In addition, electrophoretic mobility shift assays showed that Rfi2 binds to *BP1118* and *fim2* mRNAs *in vitro* (Figure S1C). ORF Finder analysis [41] of the 280-nt RNA transcribed from P_out_ predicted seven open reading frames (ORFs) composed of 12 to 22 codons, but none of the predicted peptides contained any already known protein domain, as determined by blast-p alignment [42]. Moreover, no Shine-Dalgarno sequence could be identified upstream of any of these ORFs by using the RBS calculator [43], suggesting that this transcript likely represents a non-coding RNA.

### Rfi2 expression is specific for *B. pertussis* among the classical bordetellae

Rfi2 may be specific for the *B. pertussis* lineage, as IS*481* are predominantly found in *B. pertussis* [25]. A comparison of the genome organization of the *B. pertussis* Tohama I *fim2*/*BP1118* locus with that of other *B. pertussis* isolates revealed that this region is conserved in 124 sequenced *B. pertussis* strains (Supplementary Table S3), including recent isolates D420 and B1917 collected in the USA [44] and the Netherlands [45], respectively. In contrast, other classical *Bordetella* species, such as *B. bronchiseptica* and *B. parapertussis*, lack the IS element next to *fim2* (Figure S2A). Northern blot analysis indeed showed that, in contrast to *B. pertussis* strains Tohama I, D420 and B1917, Rfi2 could not be detected in *B. bronchiseptica* 7865 and *B. parapertussis* Bpp5 (Figure S2B)*.* Thus, Rfi2 is most likely unique to *B. pertussis*, suggesting that Rfi2 may play a specific role in this pathogen.

### Rfi2 attenuates *fim2* transcript levels and decreases Fim2 production

To determine whether Rfi2 can impact *fim2* expression, we generated a mutant strain that does not produce Rfi2 (Δ*rfi2*) by deleting the complete IS element, including the P_out_ promoter (Figure 2(A)). The levels of *fim2* mRNA in the wt strain and in the Δ*rfi2* mutant were analysed by Northern blotting. As shown in Figure 2(B), *fim2* mRNA levels were strongly increased in Δ*rfi2* compared to the parental strain, while overexpression of Rfi2 from a plasmid (P*rfi2* strain) restored the expression of *fim2* to the levels seen in the wt strain (Figure 2, panels C and D).

**Figure 3.**
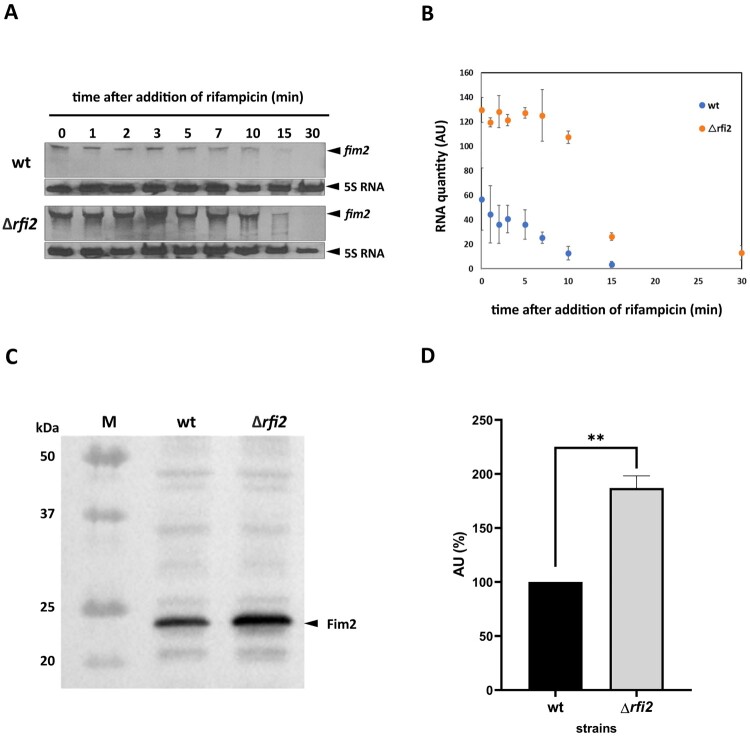
Impact of Rfi2 on *fim2* stability and translation (A) The stability of *fim2* mRNA was assayed by Northern blot in *B. pertussis* wt and Δ*rfi2* strains. Total RNA was extracted from cells harvested before (time 0) and at the indicated time points after the addition of 150 µg/ml rifampicin (1 to 30 min) and probed with biotinylated probes. The signals corresponding to *fim2* (upper blots) and to 5S RNAs (lower blots, loading controls) are indicated by black arrowheads for each strain. Only the relevant parts of the membranes are shown. The result is a representative of two independent experiments. (B) The signals obtained from two independent experiments were quantified using Scion Image software. The *fim2*-specific signals in the wt (blue) and Δ*rfi2* (orange) strains were quantified and normalized to the 5S RNA-specific signals. The normalized *fim2*-specific signals are expressed in arbitrary units (AU) and shown as means and standard deviations. (C) The production of Fim2 in the wt and Δ*rfi2* strains was assayed by Western blot analysis. Samples of cell lysates equivalent to 0.1 OD_600_ unit were separated by electrophoresis on 12.5% SDS-polyacrylamide gels and probed with anti-Fim2 antibodies. The signals corresponding to Fim2 are indicated by the black arrowhead. The protein ladder (M) with the indicated position of 25-kDa protein were loaded onto the gel along with the cell lysates. Only the relevant part of the membrane is shown. The result is a representative of two independent experiments. (D) The Fim2-specific signals detected by Western blot analysis in two independent experiments with the wt (black) and Δ*rfi2* (grey) strains were quantified using BioRad Quantity One software and expressed in arbitrary units (AU), Fim2 level in the wt strain was set to 100%. Results are shown as means and standard deviations. Differences were statistically tested with an unpaired *t*-test; **, *p* < 0.01.

To further examine the effect of Rfi2 on *fim2* expression, we determined the stability of the *fim2* transcript in wt and Δ*rfi2* cells. Exponentially grown cultures of both strains were treated with rifampicin and the cells were harvested at different time points for RNA isolation. The levels of the *fim2* transcript were determined by Northern blot analysis and quantified by scanning the *fim2*-specific signals. As shown in Figure 3(A,B), the abundance but not the stability of the *fim2* transcript was increased in the Δ*rfi2* strain compared to the parental strain.

Since Rfi2 had a negative effect on *fim2* mRNA levels, we tested how this effect translated into production of the Fim2 protein. The amounts of Fim2 in the wt and Δ*rfi2* strains were examined in biological duplicates by immunoblot analysis and quantified by using image analysis software. This analysis revealed that the amount of Fim2 in the *Δrfi2* strain was significantly increased compared to the wt strain (Figure 3(C,D)), suggesting that Rfi2-mediated attenuation of *fim2* transcript levels results in reduced production of Fim2 protein.

### The stability of Rfi2 is strongly increased in the absence of *fim2* expression

**Figure 4.**
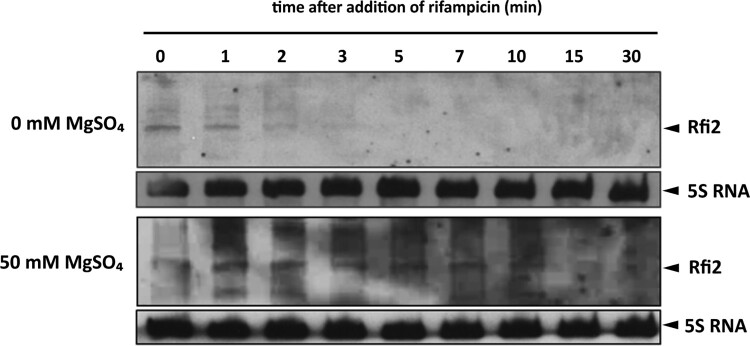
The stability of Rfi2 RNA under modulating conditions. The stability of Rfi2 RNA was determined by Northern blot analysis in wt cells cultured in modified SS medium in the absence (upper panel) or presence of 50 mM sulphate. Total RNA was extracted from cells harvested before (time 0) and at the indicated time points after the addition of rifampicin and probed with biotinylated probes. The signals corresponding to Rfi2 (upper blots) and to 5S RNAs (lower blots, loading controls) are depicted with arrowheads. Only the relevant parts of the membranes are shown. The result is a representative of three independent experiments.

RNA duplexes resulting from the interaction between mRNA and asRNA are often degraded by RNase III. As in the absence of Rfi2 the abundance of the *fim2* transcript increased, we wondered whether the absence of *fim2* would reciprocally affect the abundance and stability of Rfi2. *B. pertussis* wt cells were grown in the absence or presence of 50 mM sulphate to mid-exponential phase followed by rifampicin treatment. At different time points of rifampicin treatment, Rfi2 levels were determined by Northern blot analysis. The stability of Rfi2 increased when the cells were grown in the presence of sulphate, when *fim2* is not expressed, compared to cells grown in the absence of sulphate, when *fim2* is expressed (Figure 4).

### Rfi2 reduces cytotoxicity of *B. pertussis* towards human macrophages

**Figure 5.**
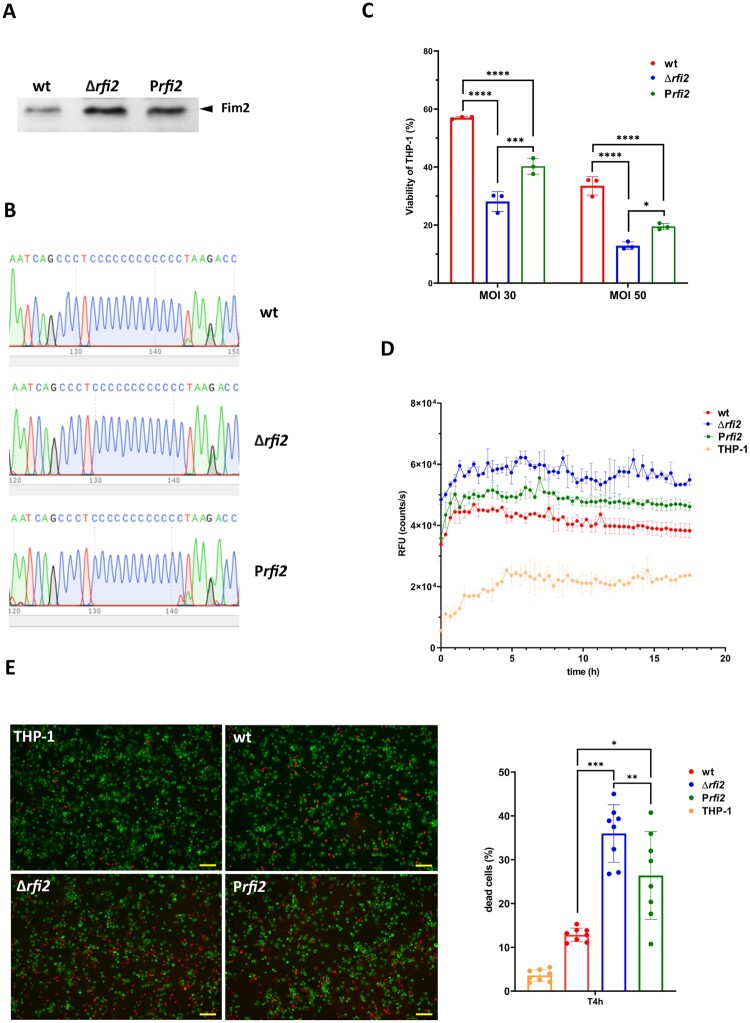
Impact of Rfi2 on the pathogenicity of *B. pertussis*. (A) The production of Fim2 in the wt, Δ*rfi2* and P*rfi2* strains was investigated by Western blot analysis. Samples of cell lysates equivalent to 0.1 OD_600_ unit were separated by electrophoresis on 12.5% SDS-polyacrylamide gels and probed with anti-Fim2 antibodies. The signals corresponding to Fim2 are indicated by the black arrowhead. The result is a representative of two independent experiments. (B) DNA sequence electropherograms showing the C-rich stretch within the promoter region of the *fim2* gene in wt, Δ*rfi2* and P*rfi2* strains. Sequences obtained from Eurofins Genomics Europe were visualized using SnapGene viewer. (C) Viability of THP-1 macrophages infected with wt, Δ*rfi2* and P*rfi2* strains. Macrophages were infected in triplicate with *B. pertussis* strains at MOIs of 30 and 50 bacteria per macrophage for 2 h at 37°C. The macrophages were then washed with fresh RPMI and finally incubated in 200 µl RPMI and 20 µl WST-1 reagent for 40 min. After incubation, the absorbance of the samples, which is proportional to the viability of the cells, was measured at 450 nm using multi-well spectrophotometer. The absorbance of uninfected cells treated in parallel in the same manner was arbitrarily set to 100%. The bars represent mean values ± standard deviation, the labels above the bars indicate the mean values of cell viability (%). Statistical analysis was performed using a two-way ANOVA test for multiple comparisons (Sidak´s test); *, *p*-value < 0.05, ***, *p*-value < 0.0005, ****, *p*-value < 0.0001. The result is representative of three independent experiments. (D) Cytotoxicity of wt, Δ*rfi2* and P*rfi2* strains towards THP-1 macrophages. Macrophages were infected in triplicate with all strains (MOI of 50). Uninfected cells served as control. Immediately after the addition of the fluorescent dye, THP-1 cells were incubated for 18 h (37°C, 5% CO_2_) in the microplate reader. During incubation, the fluorescence of the samples, which is proportional to cytotoxicity, was measured every 20 min. The graph shows the mean values and the standard errors of the means. The result is representative for two independent experiments. (E) Left; cytotoxicity of wt, Δ*rfi2* and P*rfi2* strains towards THP-1 macrophages was determined by fluorescence microscopy. In parallel with the cytotoxicity assay (panel D), infected cells and uninfected cells, which served as control, were stained with PI and SYTO-9 fluorochromes and imaged with the Olympus IX83 fluorescence microscope at 10x magnification (scale bar = 100 µm). Right; the live (green) and dead (red) macrophage cells of each strain were counted in eight different fields. Cytotoxicity is expressed as a percentage of dead cells. Statistical analysis was performed using a one-way ANOVA test for multiple comparisons (Tukey´s test). *, *p*-value < 0.05, **, *p*-value < 0.005, ***, *p*-value < 0.0001 ****.

Given the role of Fim2 in virulence of *B. pertussis* and the unique expression of Rfi2 among classical *Bordetella* species, we asked whether the Δ*rfi2* mutant exhibits a specific phenotype when interacting with human cells. Since *B. pertussis* is known to interact with human monocytes and macrophages via its fimbriae [46], we tested the effect of Rfi2 on the fate of THP-1-derived macrophages upon infection with *B. pertussis*. The overnight cultures of the wt, Δ*rfi2* mutant and the complemented P*rfi2* mutant were prior to infection examined for Fim2 levels by Western blot and for the number of cytosine residues in the *fim2* promoter region by DNA sequencing. This was done as to prove that modified production of Fim2 protein did not result from spontaneous alterations in the length of the C-rich region within the *fim2* promoter during cultivation. These analyses showed that (a) the Δ*rfi2* mutant produced increased amounts of Fim2 compared to wt, while Fim2 levels in the complemented P*rfi2* mutant were only partially reduced (Figure 5(A)) and (b) the sequence of the C-rich stretch was not altered in either strain (Figure 5(B)), indicating that the altered production of Fim2 did not result from modification of the promoter region of the *fim2* gene.

Next, we tested the cytotoxic effects of *B. pertussis* strains towards THP-1 macrophages. First, the viability of THP-1 macrophages infected with *B. pertussis* at MOI 30 and 50 was determined spectrophotometrically as the capacity of mitochondrial dehydrogenase to induce cleavage of the tetrazolium salt to formazan. Compared to macrophages infected with wt bacteria, the viability of macrophages infected with the Δ*rfi2* mutant was significantly reduced at both MOIs (Figure 5(C)). The macrophages infected with the complemented mutant overexpressing Rfi2 exhibited higher viability than the Δ*rfi2* mutant, however, the viability did not reach the value obtained with the wt strain. Second, to determine whether the reduced viability resulted from increased cytotoxic effects caused by the mutant, we measured changes in membrane integrity by quantifying fluorescence emission due to CellTox™ Green integration into the DNA of dead cells. Macrophages were infected with a MOI 50 and the fluorescence resulting from the binding of the dye to DNA in impaired macrophages was monitored in real time for up to 18 h. In agreement with the cell viability assays, the cytotoxicity caused by the mutant strain was consistently higher than that of the wt strain (Figure 5(D)), while the overexpression of Rfi2 RNA in the complemented *Prfi2* mutant reduced the cytotoxicity only partially.

In parallel with the cytotoxicity assay, infected cells and uninfected cells, which served as controls, were examined by fluorescence microscopy four hours post infection to identify viable and nonviable macrophage cells. The macrophages were stained with SYTO-9 and propidium iodide (PI) to distinguish between viable and dead cells. PI emits red fluorescence and is taken up by necrotic cells, while SYTO-9 is a green fluorochrome that stains cells with intact membranes in the presence of PI. In support of the cytotoxicity assay, nonviable (red) cells were most numerous in the sample of macrophages infected with the Δ*rfi2* mutant (36.0%) compared to the wt strain (12.9%), the complemented P*rfi2* mutant (26.4%) and uninfected cells (3.6%) (Figure 5(E)).

## Discussion

Due to the large number of IS elements in the genome of *B. pertussis* (> 240 copies), the presence of these genetic elements substantially modulates the global transcriptome profile in this pathogen [7]. Similar to other IS elements such as IS*10* of *Escherichia coli* [47,48], the primary role of P_out_ of the *BP1118* transposase gene is to regulate transposase expression to suppress transpositions and control genome rearrangements. The transcript driven by the P_out_ promoter hybridizes with the transposase mRNA, thereby destabilizing the transposase transcript and diminishing its translation [47,49]. The complementarity of 19 nt between the transcripts initiating from P_in_ and P_out_ of IS*481* is comparable to the overlap of 35 nt between the RNA-IN and RNA-OUT of *E. coli* IS*10* [40]. Given the similar configuration described here, it is likely that this antisense RNA-based mechanism is also employed by *B. pertussis* to control the activity of IS elements.

Our previous analysis of the primary transcriptome of *B. pertussis* has shown that IS*481* elements affect the expression of neighbouring genes [7]. IS elements have previously been described to affect the expression of two *B. pertussis* genes, *katA* and *bteA*, located on the same strand immediately downstream of the P_out_ promoter [30,31]. Here we describe the first IS element-driven regulatory asRNA in *B. pertussis*, Rfi2, which is transcribed in the opposite direction to the neighbouring *fim2* gene and downregulates its expression. Rfi2 does not appear as a distinct single band in a Northern blot assay. We hypothesize, that similar to the P_out_ promoter of IS*10* [39], the lack of a strong terminator downstream of the P_out_ promoter of the *BP1118* transposase gene yields several read-through transcripts of different lengths, all of which have, nevertheless, the potential to pair with the *fim2* transcript. Importantly, we found here that Rfi2 is expressed not only in the lab-adapted and highly passaged strain Tohama I, but also in recent *B. pertussis* isolates D420 and B1917. Considering that both recent isolates produce serotype 3 fimbriae, whereas Tohama I produces only serotype 2 fimbriae, this suggests that the expression of Rfi2 is not associated with a specific Fim serotype. Furthermore, the IS*481*-*fim2* locus is conserved in 124 sequenced *B. pertussis* strains but not in other classical *Bordetella* species, suggesting that the insertion of IS*481* adjacent to *fim2* occurred shortly after the *B. pertussis* lineage diverged from the *B. bronchiseptica*-like ancestor [50].

While the *fim2* gene belongs to the BvgAS regulon and its expression is negligible in the Bvg^-^ phase, the expression of Rfi2 is BvgAS-independent, but its stability is increased in the absence of *fim2* expression. Bacterial asRNAs act through various mechanisms, including transcription interference or attenuation, inhibition of translation initiation and modulation of mRNA stability [51]. The relatively large distance between P_out_ and the *fim2* promoter suggests that transcriptional interference or attenuation are unlikely mechanisms. Nevertheless, the only partial inhibitory effect of Rfi2 expressed in *trans* indicates that there may be some degree of interference between converging Rfi2 and *fim2* transcripts. However, the increased abundance of the *fim2* transcript in the Δ*rfi2* mutant and conversely the increased stability of Rfi2 in the absence of *fim2* expression rather suggest that the overlapping transcripts generate a double-stranded Rfi2-*fim2* duplex that is cleaved by RNase III. The outcome of this base pairing would largely depend on the transcription rates and, thus, on the relative abundance of both transcripts. The expression of *fim2* is maximal in the Bvg^+^ phase and it is therefore possible that most of the Rfi2 RNA bound to the *fim2* mRNA is rapidly degraded by RNase III. In contrast, in the Bvg^-^ phase, when the expression of *fim2* is minimal, only a relatively small amount of Rfi2 is engaged in the duplex and therefore the stability of Rfi2 RNA is increased.

*B. pertussis* fimbriae are generally thought to be responsible for attachment to epithelial cells, although there is limited experimental evidence for their specific role in adhesion. Both fimbriae and filamentous haemagglutinin are required for adhesion of *B. pertussis* to laryngeal epithelia cells Hep-2 [52,53], but a clear phenotype for *fim*-deficient mutants of *B. pertussis* is lacking. In the closely related *B. bronchiseptica*, fimbriae have been shown to be involved in adherence to ciliated rabbit and mouse airway epithelial cells [53,54] and to modulate the innate immune response in mice [53]. *B. bronchiseptica* cells lacking fimbriae did not adhere to the airway epithelium and localized to the alveoli, where they caused increased inflammation [53]. On the other hand, *B. pertussis* fimbriae have been shown to interact with human monocytes via binding to very late antigen-5 [46]. In this study, we show that the *Δrfi2* mutant, which produces more Fim2 than the parental strain, exhibits increased cytotoxicity to human monocyte-derived THP-1 macrophages. It should be noted that complementation of the *Δrfi2* mutant *in trans* with a plasmid overexpressing Rfi2 did not fully restore the wt phenotype and resulted an intermediate production of Fim2 and cytotoxicity. This indicates that silencing of *fim2* transcription *in trans* is not as effective as in the *cis* configuration in the wt strain. Although we do not infer that Fim2 by itself expresses cytotoxicity, it may enhance the attachment of *B. pertussis* to the macrophages and thereby facilitate the delivery of cytotoxic effectors. It has been demonstrated that the type 3 secretion system effector BteA of *B. pertussis* exhibits a much lower cytotoxic activity than BteA of *B. bronchiseptica* due to an amino acid duplication [55]. This observation was assumed as an evolutionary adaptation of *B. pertussis* to its acute infection lifestyle in the human host, which could attenuate the immune response. We hypothesize that the *B. pertussis*-specific Rfi2-mediated attenuation of Fim2 production and the resulting reduction in cytotoxicity represents another mechanism of *B. pertussis* adaptation to its human host that fine-tunes its cytotoxic effects on human macrophages. In our future experiments, it will be of importance to investigate the role of Rfi2 in *B. pertussis* using animal models.

The Rfi2-mediated attenuation of *fim2* expression in *B. pertussis* may have yet another purpose. Fim2 is a highly immunogenic protein [32,56], and targeting fimbriae by anti-Fim antibodies leads to agglutination of the bacteria [57]. Therefore, Rfi2-mediated downregulation of *fim2* expression could contribute to evasion of the immune response. In support of this hypothesis, expression of the *fim2* gene was reduced in *B. pertussis* cells recovered from infected mouse lungs [58] and in bacteria internalized by human macrophages [38,59].

In conclusion, this study identifies Rfi2 as the first IS element-driven antisense RNA that modulates virulence of *B. pertussis*. It will be of interest to study the function of other antisense RNAs that are highly abundant in this reemerging human pathogen.

## Supplementary Material

Supplementary Table S3.pdf

Supplementary_Table_S2.docx

FigS2final.tif

Supplementary Table S1.docx

Fig_S1_final.tif
