# Supplementary material for: An IS element-driven antisense RNA attenuates the expression of serotype 2 fimbriae and the cytotoxicity of Bordetella pertussis
Source: Emerg Microbes Infect. 2025 Jan 9;14(1):2451718. doi: 10.1080/22221751.2025.2451718 (PMC11774165; doi:10.1080/22221751.2025.2451718)
Supplement: Supplementary Table S3.pdf [file TEMI_A_2451718_SM7522.pdf]

Supplementary Table S3. Alignment of 300 bp region between *BP1118* and *fim2* genes

Isolate	% identity	Alignment (bp)	Mismatches	Gaps	Start	End	Orientation	E-value
Tohama I	100	300	0	0	1175755	1176054	Forward	6.4E-155
CS	100	300	0	0	1200392	1200691	Forward	6.46E-155
A371	100	253	0	0	1	253	Forward	1.88E-129
B199	99.617	261	1	0	1	261	Forward	3.65E-132
B203	99.617	261	1	0	1	261	Forward	3.63E-132
H375	100	259	0	0	4323	4581	Reverse	1.05E-132
H603	100	259	0	0	4321	4579	Reverse	1.04E-132
H617	100	255	0	0	1	255	Forward	1.57E-130
UK1_rpt	100	229	0	0	489	717	Reverse	1.96E-116
L619	100	227	0	0	506	732	Reverse	2.41E-115
FR145	100	227	0	0	1	227	Forward	2.46E-115
FR287	100	229	0	0	4312	4540	Reverse	2.02E-116
B1865	100	227	0	0	11942	12168	Reverse	2.49E-115
UK3_rpt	100	231	0	0	495	725	Reverse	1.7E-117
B1917	100	202	0	0	1	202	Forward	9.25E-102
B1920	100	195	0	0	1	195	Forward	6.32E-98
H939	99.667	300	1	0	353642	353941	Forward	2.68E-153
I002	99.667	300	1	0	411241	411540	Forward	2.69E-153
O1-CNMC-00	100	269	0	0	8519	8787	Reverse	4.07E-138
O1-SEAT-00	99.667	300	1	0	91510	91809	Forward	2.68E-153
B096	100	240	0	0	1	240	Forward	2.17E-122
SRR1610566	100	253	0	0	500	752	Reverse	1.79E-129
FR6016	99.667	300	1	0	17628	17927	Reverse	2.6E-153
FR6029	99.667	300	1	0	17630	17929	Reverse	2.59E-153
Z020	99.667	300	1	0	24	323	Forward	2.57E-153
Y397	99.667	300	1	0	17612	17911	Reverse	2.57E-153
L15249	99.667	300	1	0	24	323	Forward	2.58E-153
L15141	99.661	295	1	0	17499	17793	Reverse	1.32E-150
L15189	99.661	295	1	0	17520	17814	Reverse	1.33E-150
L14302	99.667	300	1	0	24	323	Forward	2.58E-153
L14298	99.661	295	1	0	1	295	Forward	1.33E-150
L14401	99.661	295	1	0	1	295	Forward	1.33E-150
L14394	99.661	295	1	0	1	295	Forward	1.33E-150
L15022	99.661	295	1	0	1	295	Forward	1.33E-150
L14404	99.661	295	1	0	1	295	Forward	1.33E-150
L15048	99.661	295	1	0	1	295	Forward	1.33E-150
L15023	99.663	297	1	0	17582	17878	Reverse	1.09E-151
L15080	99.667	300	1	0	17582	17881	Reverse	2.57E-153
L15053	99.667	300	1	0	17582	17881	Reverse	2.57E-153
L15690	99.661	295	1	0	17572	17866	Reverse	1.33E-150
L15592	99.661	295	1	0	1	295	Forward	1.33E-150
L15581	99.663	297	1	0	17582	17878	Reverse	1.09E-151
L15556	99.663	297	1	0	17582	17878	Reverse	1.09E-151
L15497	99.661	295	1	0	1	295	Forward	1.33E-150
L15495	99.661	295	1	0	17500	17794	Reverse	1.33E-150
L15466	99.661	295	1	0	1	295	Forward	1.33E-150
L15360	99.661	295	1	0	1	295	Forward	1.33E-150
L15803	99.661	295	1	0	1	295	Forward	1.33E-150
L15711	99.661	295	1	0	1	295	Forward	1.33E-150

L12030	99.667	300	1	0	24	323	Forward	2.58E-153
L12072	99.667	300	1	0	24	323	Forward	2.58E-153
L12152	100	250	0	0	11999	12248	Reverse	8.34E-128
L13030	99.661	295	1	0	1	295	Forward	1.32E-150
L13038	99.663	297	1	0	17590	17886	Reverse	1.09E-151
L13055	99.661	295	1	0	1	295	Forward	1.33E-150
L13091	99.663	297	1	0	17582	17878	Reverse	1.09E-151
L13118	100	250	0	0	12006	12255	Reverse	8.35E-128
L13159	99.661	295	1	0	17515	17809	Reverse	1.33E-150
L14018	99.661	295	1	0	1	295	Forward	1.33E-150
L14182	99.661	295	1	0	1	295	Forward	1.33E-150
L14184	99.661	295	1	0	1	295	Forward	1.33E-150
L14233	99.663	297	1	0	17582	17878	Reverse	1.09E-151
L14238	99.64	278	1	0	1	278	Forward	2.24E-141
L14050	99.661	295	1	0	1	295	Forward	1.33E-150
L14114	99.661	295	1	0	1	295	Forward	1.33E-150
L14149	99.663	297	1	0	17582	17878	Reverse	1.09E-151
L14179	99.661	295	1	0	1	295	Forward	1.33E-150
L15306	99.667	300	1	0	24	323	Forward	2.58E-153
L15316	99.661	295	1	0	1	295	Forward	1.33E-150
L14260	99.661	295	1	0	8521	8815	Reverse	1.32E-150
L14280	99.663	297	1	0	17582	17878	Reverse	1.09E-151
L15116	99.661	295	1	0	1	295	Forward	1.33E-150
L15121	99.667	300	1	0	17581	17880	Reverse	2.57E-153
L15101	99.661	295	1	0	17582	17876	Reverse	1.33E-150
L15108	99.661	295	1	0	17581	17875	Reverse	1.33E-150
L15221	99.667	300	1	0	24	323	Forward	2.58E-153
)AARGOS_1	99.667	300	1	0	4044040	4044339	Forward	2.73E-153
B3582	99.667	300	1	0	2895678	2895977	Reverse	2.73E-153
B3621	99.667	300	1	0	2891056	2891355	Reverse	2.73E-153
CHLA-13	99.667	300	1	0	21016	21315	Reverse	2.68E-153
CHLA-20	100	259	0	0	17549	17807	Reverse	1.08E-132
CHLA-26	99.667	300	1	0	57662	57961	Reverse	2.69E-153
FR4930	99.667	300	1	0	48	347	Forward	2.55E-153
FR4953	99.667	300	1	0	51	350	Forward	2.57E-153
FR4964	99.667	300	1	0	188	487	Forward	2.59E-153
FR5009	99.667	300	1	0	17593	17892	Reverse	2.56E-153
FR5302	99.667	300	1	0	48	347	Forward	2.56E-153
FR6005	99.667	300	1	0	17595	17894	Reverse	2.57E-153
FR6006	99.667	300	1	0	17595	17894	Reverse	2.57E-153
FR6022	99.667	300	1	0	48	347	Forward	2.61E-153
5456-P2M	99.333	300	2	0	51	350	Forward	3.15E-152
FR3469-P2M	99.667	300	1	0	51	350	Forward	2.57E-153
FR4202-P2M	99.667	300	1	0	51	350	Forward	2.57E-153
FR4624-P2M	99.667	300	1	0	17875	18174	Reverse	2.57E-153
FR4929-P2M	99.667	300	1	0	17629	17928	Reverse	2.58E-153
FR6115	99.667	300	1	0	17694	17993	Reverse	2.56E-153
FR6117	99.667	300	1	0	51	350	Forward	2.55E-153
FR6134	99.667	300	1	0	51	350	Forward	2.56E-153
FR4991	99.667	300	1	0	17601	17900	Reverse	2.56E-153
TN0006	99.667	300	1	0	51	350	Forward	2.57E-153
TN0007	99.667	300	1	0	51	350	Forward	2.57E-153

FR4808	99.667	300	1	0	17629	17928	Reverse	2.56E-153
FR5015	99.667	300	1	0	168	467	Forward	2.58E-153
FR5016	99.667	300	1	0	17629	17928	Reverse	2.56E-153
FR5258	99.667	300	1	0	231	530	Forward	2.59E-153
FR5259	99.667	300	1	0	51	350	Forward	2.57E-153
FR5731	99.667	300	1	0	49	348	Forward	2.57E-153
FR5006	99.667	300	1	0	51	350	Forward	2.57E-153
FR5013	99.667	300	1	0	51	350	Forward	2.57E-153
FR5344	99.667	300	1	0	51	350	Forward	2.56E-153
FR6395	99.667	300	1	0	51	350	Forward	2.57E-153
FR6419	99.667	300	1	0	51	350	Forward	2.57E-153
FR6424	99.667	300	1	0	51	350	Forward	2.57E-153
FR4995	99.667	300	1	0	47	346	Forward	2.57E-153
FR6576	99.634	273	1	0	1	273	Forward	1.16E-138
FR6583	99.634	273	1	0	17562	17834	Reverse	1.15E-138
FR6597	98.556	277	1	1	1	277	Forward	1.72E-136
FR6625	99.636	275	1	0	17562	17836	Reverse	9.51E-140
FR6632	99.635	274	1	0	1	274	Forward	3.32E-139
FR6648	99.634	273	1	0	1	273	Forward	1.16E-138
FR6663	99.634	273	1	0	1	273	Forward	1.16E-138
FR6807	99.634	273	1	0	1	273	Forward	1.15E-138
FR6812	99.636	275	1	0	17562	17836	Reverse	9.52E-140
B1836	99.636	275	1	0	17562	17836	Reverse	9.54E-140
