# Supplementary material for: An IS element-driven antisense RNA attenuates the expression of serotype 2 fimbriae and the cytotoxicity of Bordetella pertussis
Source: Emerg Microbes Infect. 2025 Jan 9;14(1):2451718. doi: 10.1080/22221751.2025.2451718 (PMC11774165; doi:10.1080/22221751.2025.2451718)
Supplement: Supplementary_Table_S2.docx [file TEMI_A_2451718_SM7521.docx]

**Supplementary Table S2.**

|  | **Primer/probe name** | **Sequence** |
| --- | --- | --- |
| **Cloning primers** | pSS1129 | ccccgaaaagtgccacctgac |
|  |  | cgtagatgcacatcgttgcgctc |
|  | Rfi2 | gcgattacctgtacggcaagaagc |
|  |  | ccgaccactgactacagcacc |
| **PCR primers for *fim2* promoter** | fim2rev1 | CAGGTGGTGTCGGTGATGG |
|  | fim2rev2 | GTGATGACGATGGTGCCGTC |
|  | fim2fw1 | CAGGAAGCCGCCGAAGG |
|  | fim2fw2 | GCATTGGCAGTGGTGGAC |
| **5'RACE primers** | A (Ion Torrent) | ccatctcatccctgcgtgtctccgactcag |
|  | P1T (Ion Torrent) | cctctctatgggcagtcggtgat |
|  | RNA adaptator | atgcgcgaattcctgtagaacgaacactagaag |
|  | *fim2* | atgacgcaggtggtgtcggtg |
|  | P_in_ of BP1118 | ccgcttcaggcacacaaact |
|  | Rfi2 | cctacgtcaaaaagaacggcga |
| ***In vitro* transcription primers** | *fim2* - start | taatacgactcactataggggcgtgcaggtacggatttc |
|  | *fim2* - stop | ctaggggtagaccacggaaa |
|  | BP1118 - start | taatacgactcactataggggagaaactggaaatcgccacc |
|  | BP1118 - stop | gctggaccatttcgagtcgacg |
|  | Rfi2 - start | taatacgactcactatagggtgttcatccggccgggctcc |
|  | Rfi2 - stop | catgggtgcgaacgaggcgac |
| **Northern Blot probes** | Rfi2 | gtcaaaaagaacggcgacgtcgaagccagcgccatcaccacttatgtgggtttttccgtg |
|  | *fim2* | accttggtgtgattcgggcc |
