# Supplementary figures and images for: An IS element-driven antisense RNA attenuates the expression of serotype 2 fimbriae and the cytotoxicity of Bordetella pertussis
Source: Emerg Microbes Infect. 2025 Jan 9;14(1):2451718. doi: 10.1080/22221751.2025.2451718 (PMC11774165; doi:10.1080/22221751.2025.2451718)

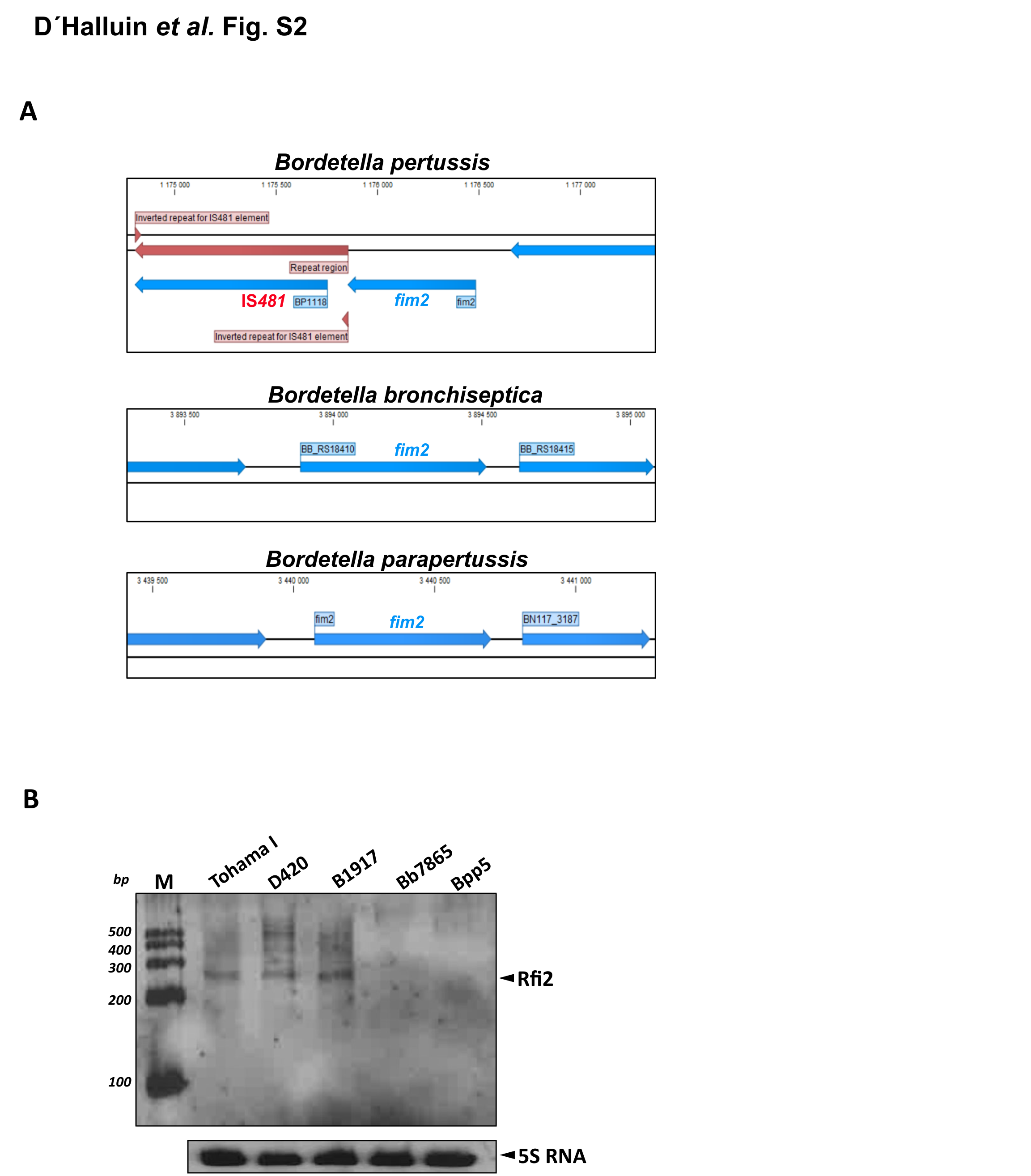

Supplement: FigS2final.tif [file TEMI_A_2451718_SM7520.tif]

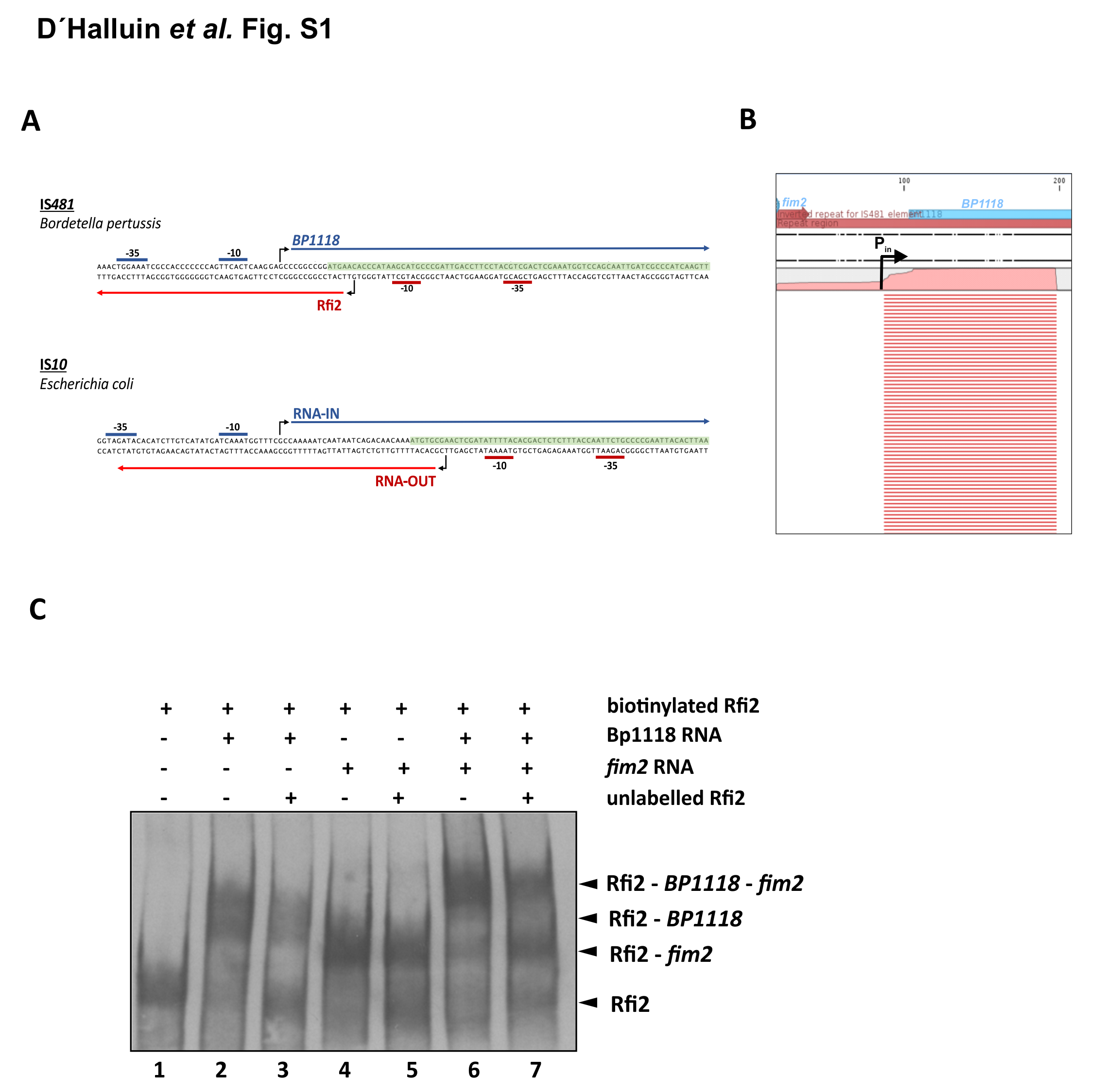

Supplement: Fig_S1_final.tif [file TEMI_A_2451718_SM7518.tif]
