# Supplementary material for: An IS element-driven antisense RNA attenuates the expression of serotype 2 fimbriae and the cytotoxicity of Bordetella pertussis
Source: Emerg Microbes Infect. 2025 Jan 9;14(1):2451718. doi: 10.1080/22221751.2025.2451718 (PMC11774165; doi:10.1080/22221751.2025.2451718)
Supplement: Supplementary Table S1.docx [file TEMI_A_2451718_SM7519.docx]

**Supplementary Table S1.**

| **Strain/plasmid** | **Description** | **Source** |
| --- | --- | --- |
| ***B. pertussis*** |  |  |
| BPSM | Derivative of Tohama I; Sm^R^ | Lab collection |
| D420 | Clinical isolate | Lab collection |
| B1917 | Clinical isolate | Lab collection |
| Δ*bvgA* | BPSM carrying in-frame deletion of the *bvgA* gene | Lab collection |
| Δ*rfi2* | Δ*BP1118*::Term2039RB50 Sm^R^ | This study |
| P*rfi2* | Δ*rfi2* strain carrying pBBR1MCS::P_out_*BP1118* | This study |
| ***B. bronchiseptica*** |  |  |
| 7865 | Human clinical isolate | CCUG, Sweden |
| RB50 | Rabitt clinical isolate | Lab collection |
| ***B. parapertussis*** |  |  |
| Bpp5 (NZ585) | Ovine isolate | Lab collection |
| ***E. coli*** |  |  |
| DH5α | Transformation strain | Invitrogen |
| TOP10 | Transformation strain | Invitrogen |
| SM10 | Conjugation strain ; RP4-2-Tc::Mu λpir ; Km^R^ | Lab collection |
| **Plasmids** |  |  |
| pJQ200mp18-rplS | Conjugation vector ; Gm^R^ | Lab collection |
| pJQ200-Δ*rfi2* | pJQ200mp18-rplS carrying the deletion of BP1118 | This study |
| pBBRMCS1 | cloning vector | Lab collection |
| pBBR1MCS::P_out_*BP1118* | pBBRMCS1 carrying the 800 bp *BP1118-fim2* fragment | This study |
| pUCIDT-KAN-Δ*rfi2* | Vector construct carrying the deletion of Δ*rfi2* | IDT |
| pCR2.1-TOPO | Cloning TA vector ; Km^R^ Am^R^ | Invitrogen |
| pCR-BP*rfi2* | pCR2.1-TOPO carrying the *rfi2* gene | This study |
| pCR-BP1118 | pCR2.1-TOPO carrying the 5’ sequence of BP1118 | This study |
| pCR-*fim2* | pCR2.1-TOPO carrying the 3’ sequence of *fim2* | This study |
